# Severity assessment of COVID-19 disease: radiological visual score versus automated quantitative CT parameters using a pneumonia analysis algorithm

**DOI:** 10.3389/fmed.2025.1606771

**Published:** 2025-09-09

**Authors:** Dario Baldi, Bruna Punzo, Andrea Colacino, Marco Aiello, Monica Franzese, Ludovico La Grutta, Luca Saba, Eduardo Bossone, Emanuela Passaro, Cesare Mantini, Carlo Cavaliere, Sergio Berti, Simona Celi, Antonella Meloni, Filippo Cademartiri, Erica Maffei

**Affiliations:** ^1^IRCCS SYNLAB SDN, Naples, Italy; ^2^Department of Radiology, University of Palermo, Palermo, Italy; ^3^Department of Radiology, University of Cagliari, Cagliari, Italy; ^4^Department of Public Health, Department of Translational Medical Sciences, University of Naples “Federico II”, Naples, Italy; ^5^Department of Radiology, University of Chieti, Chieti, Italy; ^6^Department of Interventional Cardiology, Fondazione Monasterio/CNR, Massa, Italy; ^7^Department of Bioengineering, Fondazione Monasterio/CNR, Pisa, Italy

**Keywords:** CT, pneumonia, AI, chest, COVID-19

## Abstract

**Objective:**

Evaluate the impact of the use of an AI-assisted quantitative tool for assessing stratification of patients with acute lung involvement from coronavirus (COVID-19) compared to a semi-quantitative visual score made by the radiologist.

**Methods:**

We retrospectively enrolled 611 patients with respiratory distress and suspected pneumonia admitted between 27 February and 27 April 2020. Demographic, imaging, and clinical data were collected. Lung involvement was visually assessed using a 5-class severity scale and compared with automated AI-based CT analysis (CT Pneumonia Analysis 2.5.2, SyngoVIA Siemens), which quantified volume and density of alterations. Patients were assigned to severity classes for concordance analysis. Subgroup analysis across biweekly periods assessed changes in visual rater performance. Correlation with SpO₂ and diagnostic performance (accuracy, sensitivity, specificity, AUC) of both methods in predicting RT-PCR results were evaluated.

**Results:**

High concordance was found between visual and quantitative assessments (*k* = 0.73, *p* < 0.001), with most discordances in low-severity (classes 0–1, *k* = 0.71), while agreement was excellent for higher severity (classes 2–4, *k* = 0.91). Misclassifications were mainly for mild cases; concordance was strong in severe, life-threatening presentations. Temporal analysis showed a progressive improvement in agreement over time (*k* = 0.62, 0.61, 0.54, 0.73). A mild but significant negative correlation emerged between quantitative assessment and SpO₂ values (*r* = −0.13, *p* = 0.02). Diagnostic performance between the two methods was similar: visual (AUC = 0.55, Accuracy = 44%, Sensitivity = 27%, Specificity = 78%) and quantitative (AUC = 0.56, Accuracy = 45%, Sensitivity = 27%, Specificity = 79%). Neither method showed strong predictive power for RT-PCR COVID-19 positivity. Nonetheless, assessing lung involvement remains essential for managing respiratory distress, regardless of confirmed infection, particularly for identifying patients with >25% parenchymal involvement who may require hospitalization.

**Conclusion:**

Visual and AI-based CT assessments showed high concordance and similar accuracy, especially in patients with >25% lung involvement. This study demonstrates the utility of AI-based algorithms to improve the diagnostic efficiency and the reliability, highlighting their value in routine COVID-19 pneumonia evaluation and management.

## 1 Introduction

Coronavirus disease (COVID-19) is a viral respiratory illness caused by severe acute respiratory syndrome coronavirus 2 (SARS-CoV-2) that has affected millions of individuals worldwide. COVID-19 primarily manifests as an acute respiratory infection with varying degrees of severity, ranging from asymptomatic or mild to severe and life-threatening (1–5).

To date, there is no consensus on the optimal imaging approach for the assessment of COVID-19 pneumonia. Indeed, the use of chest CT scans for the diagnosis and management of this condition has been the subject of intense debate (6). The radiological features of COVID-19 pneumonia, such as ground-glass opacities, consolidation, and bilateral pulmonary infiltrates, provide critical insights that guide clinical decisions (7). These features, detectable on CT scans, have proven invaluable in the early detection of the disease, particularly in cases where traditional testing methods like RT-PCR have limitations in sensitivity and availability (8). The significance of CT scans extends beyond diagnosis (9), enabling healthcare providers to triage patients effectively, identifying those who require immediate medical intervention from those with milder symptoms (10). While there is no doubt that chest CT scans can provide valuable information on the extent and severity of pulmonary involvement in COVID-19 pneumonia, the optimal approach for image interpretation is less clear. Two different approaches have been suggested: quantitative assessment and semi-quantitative visual scoring. The former involves the measurement of specific radiological features, such as ground-glass opacities and consolidation, and the calculation of a score based on their extent and distribution. The latter relies on visual assessment of the overall extent and distribution of pulmonary involvement, typically using a scoring system ranging from 0 to 4.

Previous studies have demonstrated the potential utility of chest CT scans in the diagnosis and management of COVID-19 pneumonia. For example, Li et al. (11) reported that chest CT scans were more sensitive than reverse transcriptase-polymerase chain reaction (RT-PCR) for the diagnosis of COVID-19 in a cohort of 1,014 patients in Wuhan, China. Similarly, Fang et al. (12) demonstrated that chest CT scans can help to differentiate between COVID-19 pneumonia and non-COVID-19 pneumonia, with ground-glass opacities being more common in the former. In addition, some studies have suggested that chest CT scans may be useful for prognostication in patients with COVID-19 pneumonia, with more extensive pulmonary involvement being associated with poorer outcomes (6, 13). In the context of COVID-19, AI-assisted CT analysis has been explored as a means to support radiologists in assessing lung involvement, particularly in settings with limited expert availability or high patient loads. The tremendous strain that COVID-19 put on the healthcare system underscored the potential benefits of AI-driven diagnostic tools in improving efficiency and accuracy. AI has the capability to rapidly and objectively quantify disease burden, potentially reducing variability and inter-observer differences inherent in visual scoring methods.

Recent studies have highlighted the potential of deep learning and radiomics-based approaches to enhance the diagnostic and prognostic utility of chest CT scans in COVID-19 and other pulmonary diseases, supporting faster and more reproducible evaluations (14, 15).

Moreover, AI-based algorithms can assist in predicting disease progression, enabling clinicians to make more informed decisions regarding patient management and resource allocation.

However, there is limited data on the comparative accuracy of semi-quantitative visual scoring and quantitative assessment of chest CT scans for the stratification of patients with COVID-19 pneumonia. Some studies have suggested that quantitative assessment may be more accurate than semi-quantitative visual scoring, due to the potential for inter-observer variability in the latter approach. For example, Yang et al. (16) reported that quantitative assessment had higher inter-observer agreement and better diagnostic accuracy than semi-quantitative visual scoring in a cohort of 72 patients with COVID-19 pneumonia. On the other hand, other studies have reported the opposite, with semi-quantitative visual scoring being more accurate than quantitative assessment. For example, Wu et al. (13) reported that visual scoring had higher diagnostic accuracy than quantitative assessment in a cohort of 74 patients. Both assessment methods play crucial roles in the clinical management of COVID-19 pneumonia, offering complementary insights. The choice between semi-quantitative and quantitative assessments can depend on various factors, including the specific needs of patient management, the availability of resources, and the objectives of clinical or research studies. Understanding the strengths and limitations of each method is essential for leveraging their benefits while mitigating potential downsides, ultimately aiming to enhance patient care and outcomes in the challenging context of COVID-19 (17).

The main aim of this study was to evaluate the effectiveness of an AI-assisted quantitative CT tool in assessing lung involvement in COVID-19 patients compared to a traditional semi-quantitative visual score assigned by radiologists. We hypothesized that quantitative assessment would be more accurate than semi-quantitative visual scoring, given the potential for inter-observer variability in the latter approach. The results of this study will have important implications for the management of patients with COVID-19 pneumonia, particularly with respect to the interpretation of chest CT scans and the usefulness of AI-based approaches on the diagnostic workflow. By comparing the two approaches, we seek to determine whether AI can enhance the accuracy and efficiency of disease severity assessment, ultimately improving patient stratification and management.

## 2 Materials and methods

### 2.1 Study population

**Figure 1:**
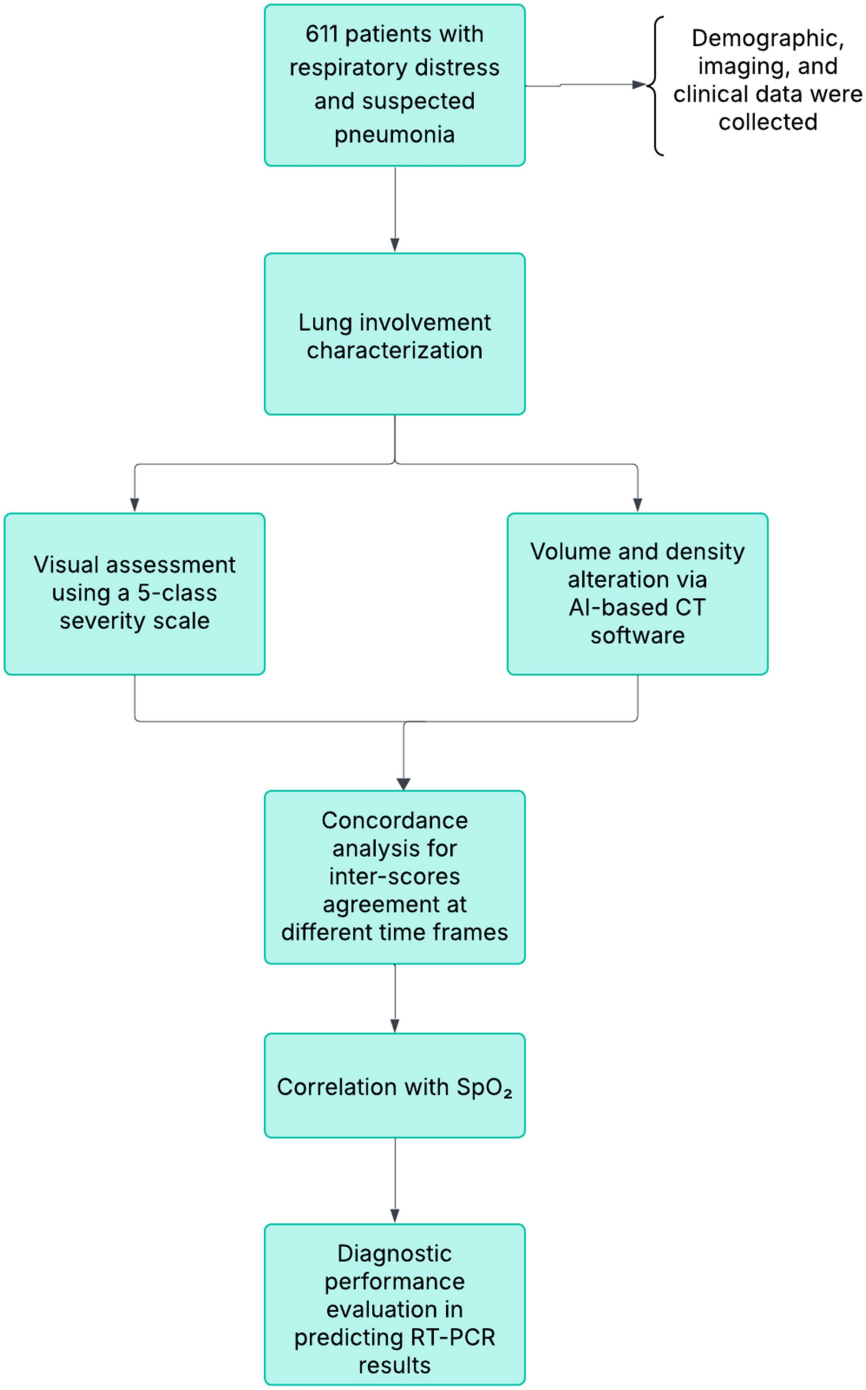
Experimental layout.

This study was a retrospective single-center study on patients enrolled during the first wave of Italy pandemic, from 27 February 2020 to 27 April 2020. Included patients were hospitalized for the suspicion of a novel coronavirus infection and underwent both chest CT imaging and laboratory virus nucleic acid testing (reverse transcription-polymerase chain reaction RT-PCR assay with nasopharyngeal and oropharyngeal swab samples). A total of 611 (mean age, 63yo; age range, 18–93; 65% men and 35% women) chest examinations CT in the initial emergency department assessment for suspected COVID were included retrospectively. For the 611 patients, saturation was measured during chest CT for 399 patients. All patients with positive RT-PCR results for COVID-19 were identified (*n* = 435). In a case with multiple swabs, the patients were rated positively if a minimum of one of the specimens was positive. Of the 611 patients, biographical data, imaging characteristics, laboratory tests, and clinical data were collected (Table 1). The experimental design has been outlined in Figure 1.

**Table 1:** Demographic characteristics and risk factors of population.

Parameter	*N* (%) or mean ± SD
Number of patients	611
Age (years)	63 ± 19
Sex (male)	(397) 65%
Height (cm)	169 ± 9
Weight (kg)	78 ± 19
Saturation	96 ± 7
FC (bpm)	85 ± 17
Smoker	(24) 3.96%
Ex-smoker	(40) 6.54%
Fever (yes)	(446) 73%
Cough (yes)	(287) 47%
Dyspnoea (yes)	(224) 36.7%
Asthenia (yes)	(41) 6.7%
Chest pain (yes)	(33) 5.4%
Arthralgias (yes)	(41) 6.7%
Dysgeusia (yes)	(34) 5.7%
Anosmia (yes)	(28) 4.6%
Diarrhea (yes)	(36) 5.9%
Shivers (yes)	(24) 3.9%
Pharyngodynia (yes)	(28) 4.6%
Headache (yes)	(38) 6.2%
Hemoptysis (yes)	(7) 1.2%
Rhinitis (yes)	(14) 2.3%
Conjunctivitis (yes)	(10) 1.6%
Nausea (yes)	(42) 6.9%

### 2.2 CT scanning protocol

All images were obtained using the Revolution EVO CT system (GE Healthcare, Milwaukee, WI, United States) with patients placed in the supine position. All the scans were performed without contrast agent administration. Scanning parameters were 120 kVp, 40–90 mAs, pitch 1–1.25, matrix 512 × 512. All images have been reconstructed with a slice thickness of 1.25 mm.

### 2.3 Image analysis

Visual chest CT interpretation was independently performed by three radiologists (E. M, F. C, and C. C) blinded to clinical data, respectively, with 15, 20, and 12 years of experience.

Each observer systematically assessed typical pulmonary abnormalities including ground-glass opacity (GGO), crazy paving patterns, and consolidations. Additional radiological signs such as emphysema, pulmonary fibrosis, nodular formations, sub-pleural linear opacities, atelectasis, and tree-in-bud patterns. In addition, for each lobe, observers graded the abnormalities using the following semiquantitative visual scoring system. Severity thresholds were defined as follows: score 0, <10%; score 1, 10–25%; score 2, 26–50%, score 3, 51–75%, score 4, greater than 76% of pulmonary parenchymal involvement.

In parallel, the CT pneumonia analysis prototype based on the AI algorithm (Platform Frontier, SyngoVIA, Siemens Healthineers, Erlangen, Germany) was used to automatically detect and quantify pathological lung findings. The system employs convolutional neural networks (CNNs) trained on an extensive and annotated datasets of 9,749 chest CT scans from patients with various lung conditions, including COVID-19 pneumonia (18), to recognize radiological patterns typical of COVID-19 pneumonia, such as ground-glass opacities, consolidations, and septal thickening. The algorithm automatically segments the lung parenchyma, identifies several radiological patterns and calculates key quantitative metrics, including: the total lesion volume relative to the overall lung volume, the quantitative density values and the percentage of lung involvement. Furthermore, based on predefined thresholds of affected lung volume, the algorithm categorizes disease severity based on the extent of lung involvement, allowing direct comparison with the radiologists’ visual assessment. The prototype processes each CT scan in approximately 80! 10 s per patient and generates an output report with the volume and opacity percentages calculated for individual lobes and for the entire lung volume. Following, these AI-based classifications were directly compared to radiologists’ semiquantitative scores to evaluate the level of concordance between manual and automated assessments. For this purpose, we grouped CT continue percentages into discrete severity score (0–4), encompassing for score 0, normal to <10%; score 1, 10–25%; score 2, 26–50%, score 3, 51–75%, score 4, >76% involvement.

### 2.4 Statistical analysis

To conduct the statistical analysis using IBM SPSS Statistics software, we utilized various techniques including weighted k-Cohen analysis for CT scoring concordance, correlation analysis with Pearson’s test, and ROC curve analysis.

Firstly, we performed a weighted k-Cohen analysis to examine the concordance between semi-quantitative visual score versus automated quantitative assessment. This analysis allowed us to assess whether there were statistically significant differences in the assessment of different severity scores. Moreover, the same approach was implemented subdividing the sample according to four enrollment time frames of 15 days to highlight the weight of training and radiologists confidence in the pandemic period. To assess statistical power for subgroup analysis, we applied a one-way ANOVA framework with α = 0.05 to detect differences across four-time groups, based on the observed variability and confidence interval widths, a standard deviation of 0.15 (assumed for Cohen’s *κ* and considering a between-group difference of Δκ ≈ 0.10 as clinically meaningful).

Secondly, we employed ANOVA to detect differences among visual and quantitative scores for SpO2 values. Moreover, a correlation analysis using Pearson’s test was used to explore the relationships between CT imaging impairment (quantitative score) and SpO2 percentage. This analysis enabled us to examine the strength and direction of the linear association between the variables. The Pearson correlation coefficient (r) was calculated, and its significance level was assessed to determine whether the observed relationship was statistically significant (*p* ≤ 0.05).

Lastly, we conducted ROC curve analysis to evaluate the predictive performance of a diagnostic test. This analysis involved plotting the true positive rate (sensitivity) against the false positive rate (1-specificity) at various classification thresholds, based on a logistic regression model, and using default parameters. The area under the ROC curve (AUC) was calculated, with values closer to 1 indicating a higher discriminatory power of the test.

## 3 Results

**Figure 2:**
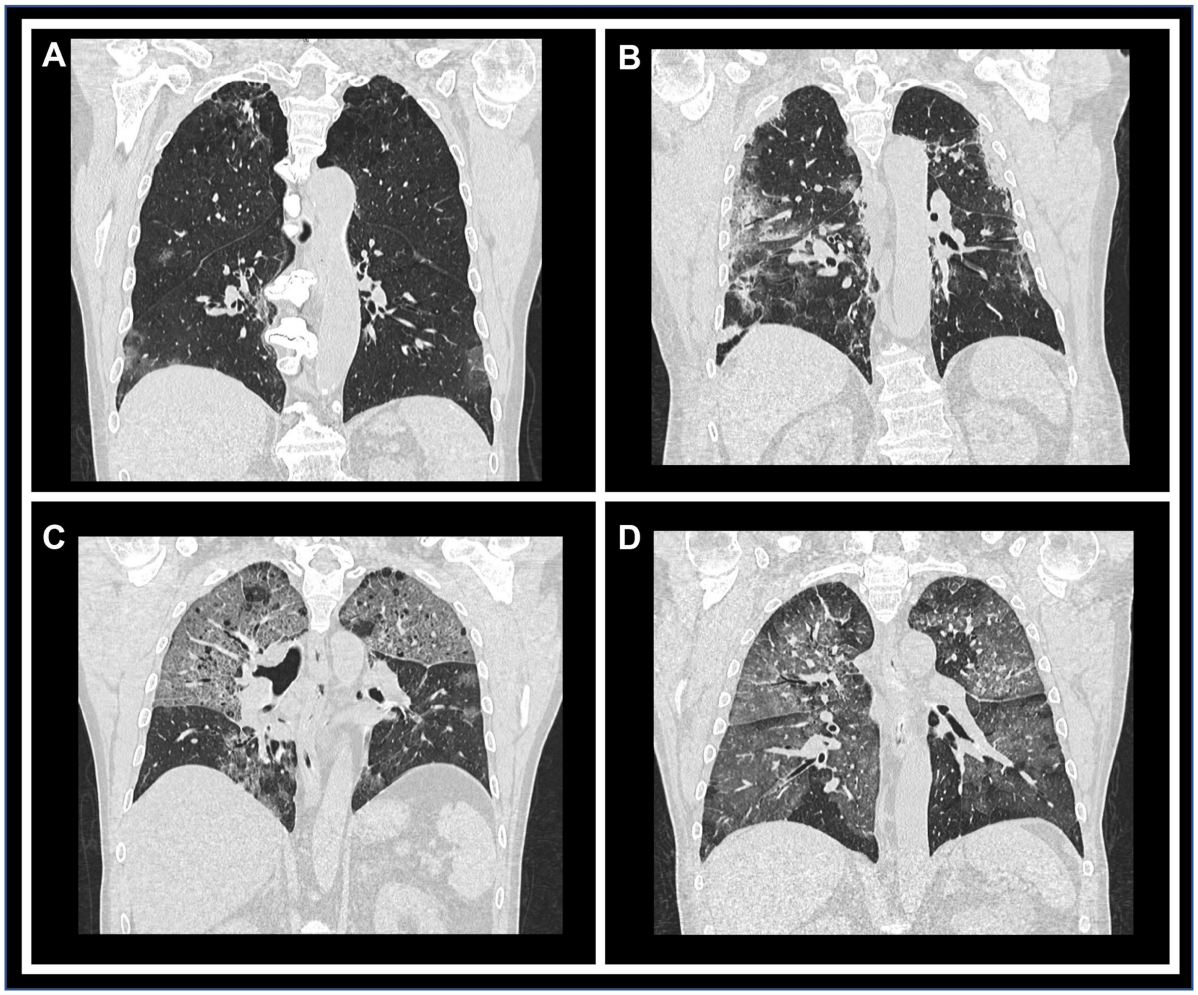
Representative CT images illustrating each qualitative/semi-quantitative scoring category. Coronal reconstructions of chest CT scans demonstrating varying degrees of parenchymal involvement. Panels **(A)** through **(D)** correspond to scores of 1 (10–25% involvement), 2 (26–50%), 3 (51–75%), and 4 (>76%), respectively, highlighting the progressive extent of lung impairment.

**Figure 3:**
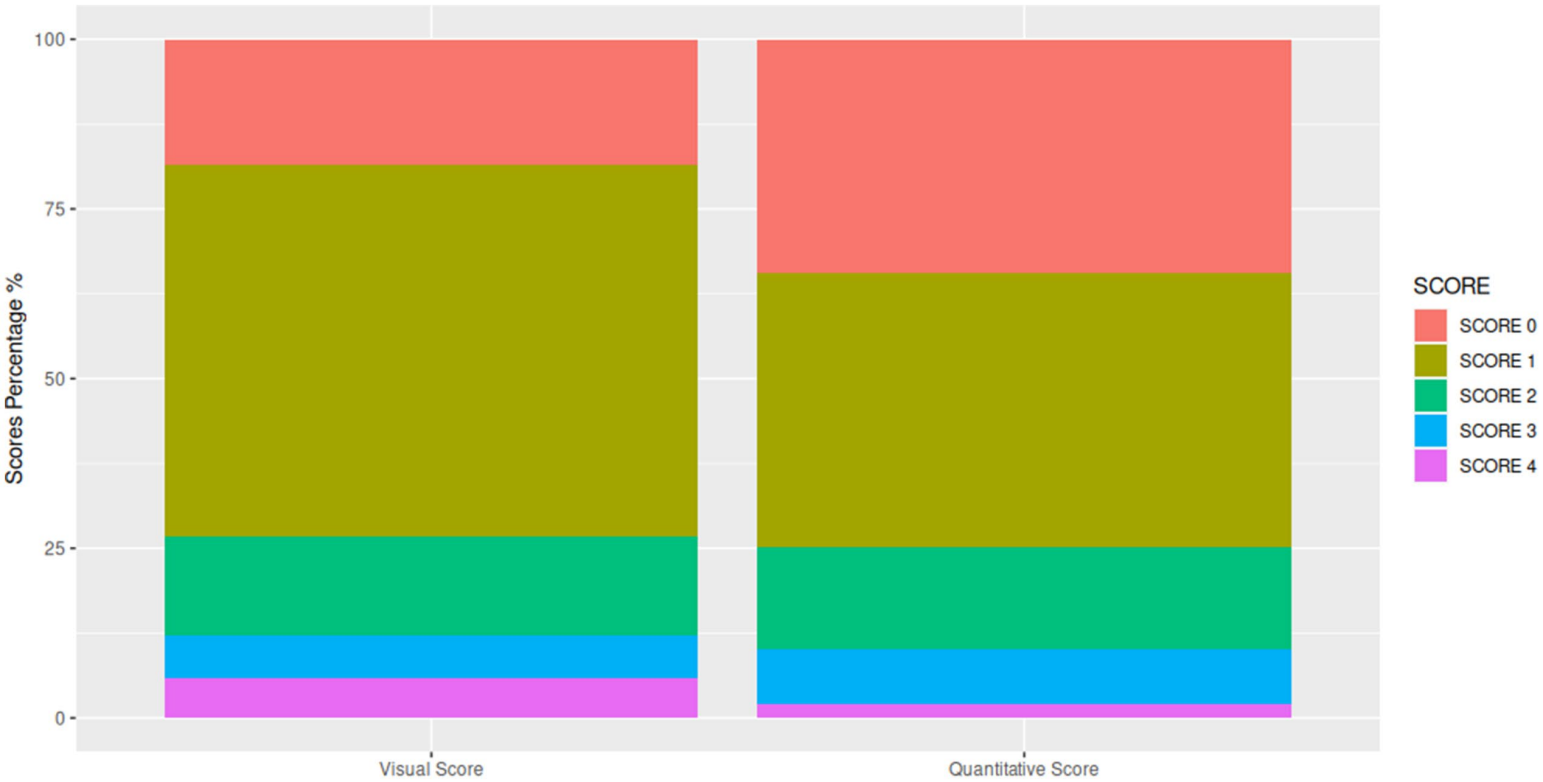
Comparative distribution of scores from visual and quantitative assessments. Bar graphs depict the percentage of cases assigned scores 0 through 4 using both the visual and quantitative scoring methods, allowing direct comparison of score frequency across the two evaluation approaches.

The analysis of the study data revealed several significant findings (Figure 2). Firstly, there was a good concordance between two methods used (*k*: 0.73, *p* < 0.001), indicating a strong relationship between qualitative and semi-quantitative measures. However, it was observed that the major source of discordance occurred for scores 0 and 1 (*k*: 0.71, *p* < 0.001), while higher classes and parenchymal involvement (Class 2–4) showed a higher level of agreement (*k*: 0.91, *p* < 0.001) (Figure 3).

**Figure 4:**
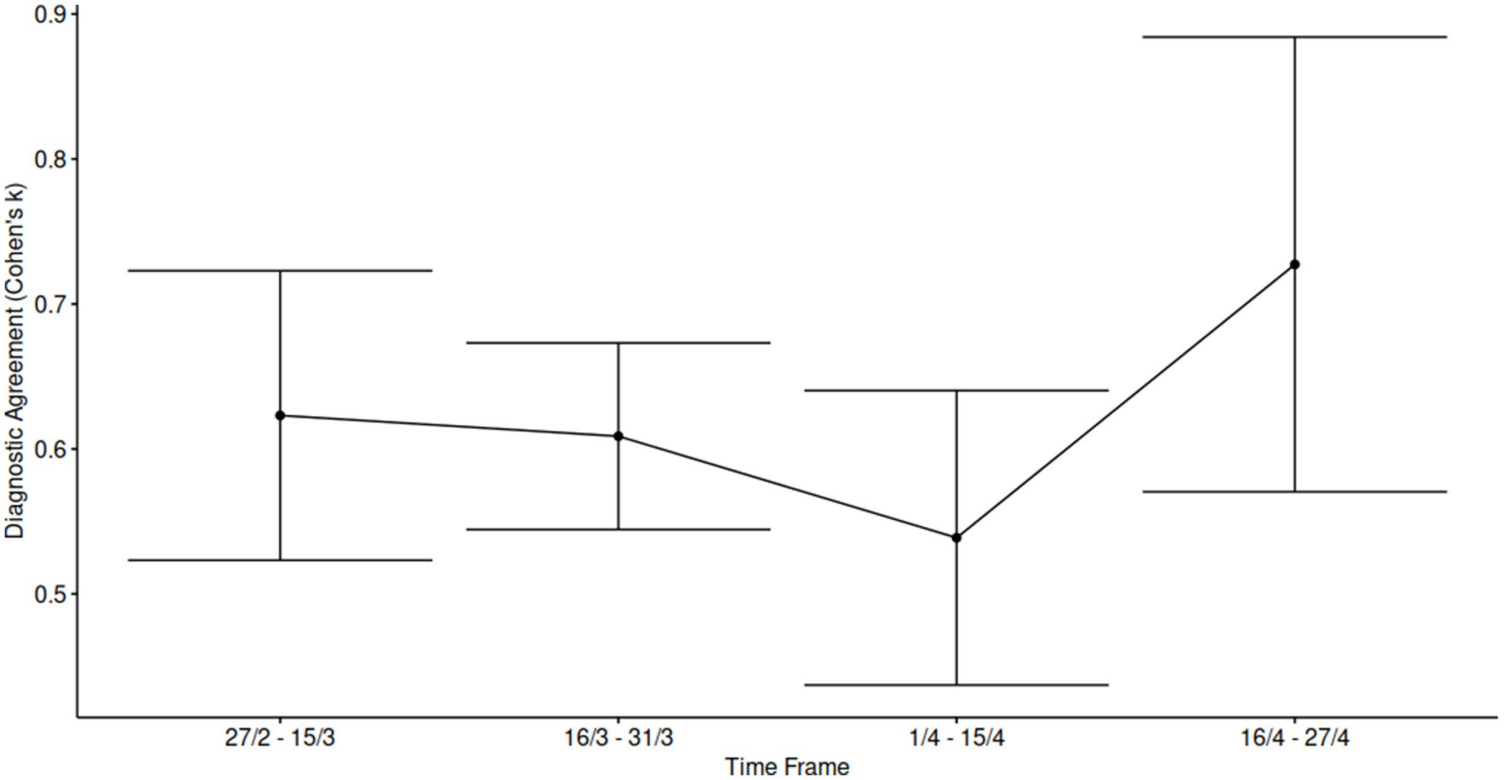
Temporal analysis of inter-rater agreement across the study period. Cohen’s kappa statistics were calculated within approximately biweekly intervals throughout the enrollment period to assess the consistency of scoring over time and potential training effects during a challenging evaluation phase. Each data point represents the mean *κ* value for the corresponding time frame, with error bars indicating 95% confidence intervals, reflecting variability in agreement levels across intervals.

Furthermore, we conducted a subgroup analysis over different timeframes, after performing a *post hoc* power assessment, (power > 0.8, α = 0.05). We considered four-time frames (sample sizes 122, 281, 166, and 42 cases, respectively). The results indicated a positive trend for concordance between the two estimations across the entire sample. The kappa values were found to be 0.62, 0.61, 0.54, and 0.73 for the four time periods studied, suggesting a moderate to substantial level of agreement over time (Figure 4).

**Figure 5:**
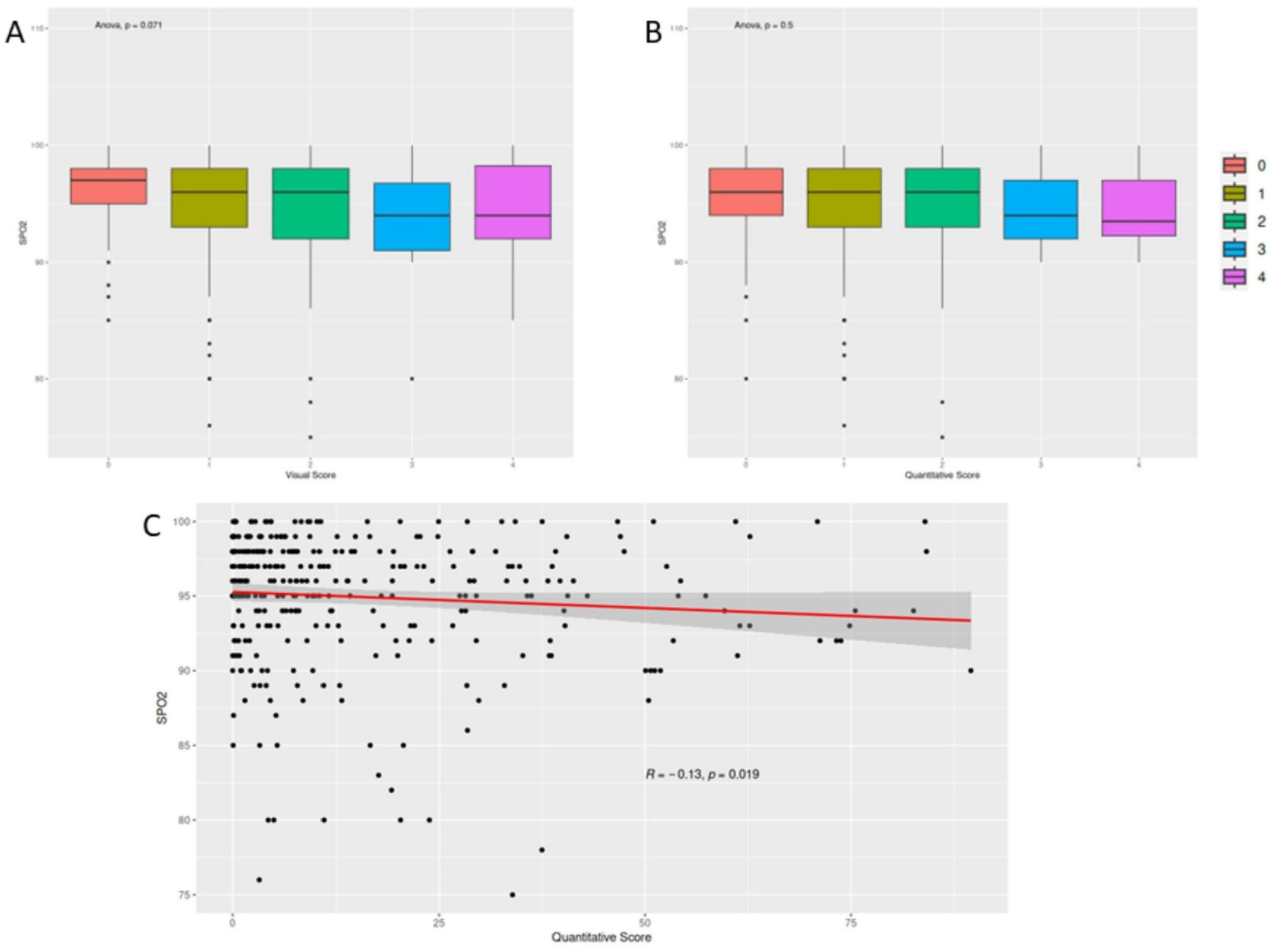
Correlation between scoring metrics and peripheral oxygen saturation (SpO2). Panels **(A,B)** present box plots comparing SpO2 values across different visual and quantitative score categories, respectively, showing no statistically significant differences. Panel **(C)** illustrates a scatter plot demonstrating a mild inverse correlation between quantitative CT scores and SpO2 levels.

Moreover, we firstly investigated differences among different scores for SpO2 values (Figures 5A,B). Following, we correlated quantitative score with peripheral oxygen saturation (SpO2%), demonstrating a significant mild anticorrelation for the quantitative assessment (*r*: −0.13, *p* value 0.019; Figure 5C).

**Figure 6:**
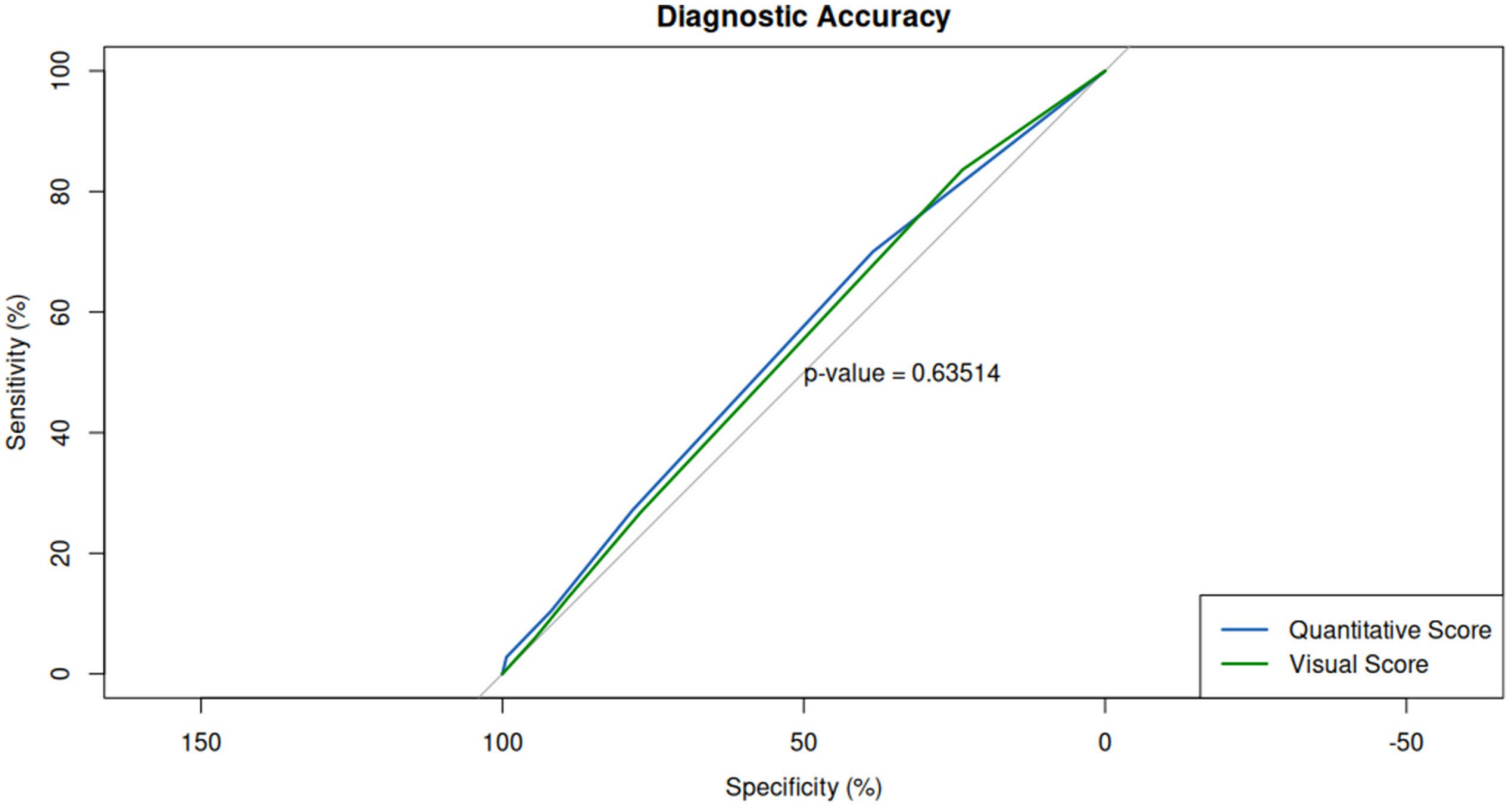
Diagnostic performance of visual and quantitative scores relative to RT-PCR swab positivity. Receiver operating characteristic (ROC) curves for both scoring methods reveal comparable diagnostic accuracy, with area under the curve (AUC) values of 0.55 for visual assessment and 0.56 for quantitative assessment, indicating no significant difference in their ability to predict swab positivity.

In terms of diagnostic performance, no significant differences were observed between the two methods compared to swab positivity. The area under the ROC curve (AUC) was 0.55 for visual assessment and 0.56 for quantitative assessment (Figure 6). The accuracy (Acc), sensitivity (Se), and specificity (Sp) values were also comparable for both methods, with visual assessment yielding an accuracy of 44%, sensitivity of 27%, and specificity of 78%, while quantitative assessment showed an accuracy of 45%, sensitivity of 27%, and specificity of 79%.

## 4 Discussion

The use of chest CT scans in the evaluation of COVID-19 pneumonia patients has become increasingly important due to its high sensitivity and ability to detect early signs of disease progression and following affecting patients’ management. However, the optimal method of CT assessment remains unclear, considering that different assessment approaches come each with its advantages and challenges. Generally, visual scoring provides a rapid and intuitive method for estimating lung involvement based on radiological patterns but is inherently subjective and prone to inter-observer variability (19, 20). In contrast, quantitative AI-based assessment offers a more objective and reproducible evaluation by generating precise volumetric measures of pulmonary alterations, although it may require technical resources and can miss subtle clinical nuances best recognized by expert radiologists.

In this study, we compared the accuracy of semi-quantitative visual scoring and quantitative assessment of chest CT scans for the stratification of patients with COVID-19 pneumonia, observing an excellent overall concordance (*κ* = 0.73), particularly in moderate to severe cases (classes 2–4, κ = 0.91), confirming the robustness of AI-assisted tools for identifying patients with substantial pulmonary involvement. However, the use of a pretrained commercial AI tool (Syngo. Via, Siemens), whose training dataset and model specifications have not been disclosed, could introduce biases. Limited transparency restricts full assessment of generalizability and performance consistency. To address these limitations, future studies will consider the use of open-source or customizable AI tools with transparent architecture and accessible training data. Additionally, external validation on multicenter datasets and with different CT acquisition protocols will be essential to assess reproducibility and improve the robustness of AI-driven quantification.

Our results showed an excellent correlation between the two methods, with a major source of discordance, although high, for scores 0 and 1 (parenchymal alterations involving less than 25%), compared to higher classes and parenchymal involvement (Class 2–4). While the rate of discordance for scores 0–1 seems to be of little influence, given the lower pulmonary involvement and thus the lower clinical severity of the patient, it is important to point out that the AI system correctly identifies patients with an extension greater than 25 percent, i.e., those that from triage should go for further diagnostic investigation because they are more at risk for respiratory complications. Our findings are consistent with previous studies that have shown a strong correlation between visual and quantitative assessment methods in assessing the extent of lung involvement in patients with COVID-19 pneumonia (21, 22). However, our study is unique in that it included a temporal subgroup analysis, which identified a positive trend for concordance between the two measures over time (k 0.62, 0.61, 0.54, 0.73 for the four-time lapse periods). This temporal subgroup analysis provided valuable insight into the evolution of inter-rater agreement (Cohen’s *κ*) during a dynamic enrollment period. Notably, it enabled us to monitor diagnostic consistency across different operational phases and to potentially detect training effects or workflow changes over time. The largest time frame (16–31 March) showed stable *κ* values with narrow confidence intervals, indicating robust statistical reliability and reflecting good calibration among raters during that period. However, one key limitation lies in the final time frame (16–27 April), which included a small sample size. While this group exhibited the highest observed κ, the wide confidence intervals indicate considerable uncertainty. This limits the reliability of the observed increase and reduces statistical power, warranting cautious interpretation of this result.

Additionally, no differences have been detected among different scores and SpO2. A mild negative correlation between quantitative assessment and SpO2 values (*r*: −0.13, *p* = 0.019), which is consistent with previous studies showing a weak association between the extent of lung involvement and the severity of hypoxemia (23, 24). A correlation for visual scores and blood oxygen saturation was not possible, considering the discrete entity of visual assessment, compared to the finer quantification of AI assessment. Finally, our ROC curve analysis showed comparable performance in terms of accuracy, sensitivity, and specificity between the two methods (mean AUC: 0.55), which is consistent with previous studies that have shown no significant difference between the two methods (25).

The mild anticorrelation between chest CT impairment and SpO2 rates demonstrated how CT morphological criteria cannot fully explain clinical pulmonary dysfunction, that have taken into account also other clinical and instrumental variables, such as age and/or D-dimer concentrations (13). Moreover, the low values of AUC for both approaches, highlighted as the chest CT findings, did not parallel to COVID-19 positivity demonstrated by RT-PCR, but identified only a specific feature in a poly-symptomatic disease involving multiple anatomical regions and domains (12). Beyond the actual impact of CT assessment in COVID patient management, the results of our study help to frame the role of AI-based tools, clearly demonstrating their ability to support the radiologist in quantifying alterations in lung CTs, lightening the workload under emergency and stress conditions (16).

The integration of AI into the diagnostic process, particularly in interpreting CT scans for COVID-19, marks a transformative shift in medical imaging and patient care. AI’s ability to rapidly process and analyze vast amounts of imaging data with high precision means it can identify patterns indicative of COVID-19 pneumonia, potentially even before these signs are clearly evident to human observers (26). This technology not only enhances the accuracy of diagnoses but also significantly reduces the time healthcare professionals spend analyzing scans. By automating the detection and assessment of pathological features in CT images, AI supports timely and accurate diagnosis, facilitating early intervention and appropriate treatment planning (27). Moreover, AI tools can manage and prioritize diagnostic workflows, ensuring patients with the most severe pathology are attended to promptly. Consequently, AI’s contribution to diagnostic radiology in the context of the COVID-19 pandemic alleviates the strain on medical staff, optimizes resource allocation, and improves patient outcomes by enabling faster and more precise diagnosis. This integration underscores the evolving synergy between technology and healthcare, promising to reshape the future of disease diagnosis and management (28).

Validating AI-based tools against traditional assessment methods in the clinical diagnosis of COVID-19 through CT scans is paramount to their successful integration into healthcare systems. This validation process is critical to ensure that AI tools not only match but potentially exceed the accuracy and reliability of human-driven assessments. Through rigorous clinical trials and comparative studies, AI algorithms are evaluated for their ability to accurately identify and quantify pathological features associated with COVID-19, such as ground-glass opacities and consolidation patterns. Validation studies, like the one conducted by Ko et al. (26), focus on various metrics, including sensitivity, specificity, and predictive values, to gage how well AI tools can detect COVID-19 cases compared to gold-standard diagnostic methods.

The necessity of this validation process stems from the need to establish a strong foundation of trust in AI technologies among healthcare professionals. By demonstrating that AI can work alongside radiologists, enhancing their capabilities rather than replacing them, it encourages wider adoption of these technologies in clinical practice. Moreover, aligning AI tools with clinical outcomes ensures that they contribute positively to patient care, aiding in the early detection and monitoring of disease progression, which is crucial for timely intervention and treatment planning. In essence, the validation of AI-based tools against traditional methods is not just a technical necessity but a clinical imperative. It ensures that the integration of AI into healthcare workflows translates into tangible benefits for patient management and outcomes. As AI continues to evolve, ongoing validation and recalibration based on real-world clinical data will be essential to maintain its relevance and efficacy in the ever-changing landscape of medical diagnostics (29–31).

In conclusion, clinically, their findings showed that AI tools can reliably identify patients with parenchymal involvement greater than 25%, a critical threshold for triage decisions. In emergency settings, this could support the prioritization of patients at higher risk of respiratory complications, enhancing the efficiency of resource allocation and care planning. In this context, AI-based quantitative CT analysis may serve as a decision-support system, particularly where rapid, standardized assessment is needed, or radiological expertise is limited.

## Data Availability

The raw data supporting the conclusions of this article will be made available by the authors, without undue reservation.

## References

[1] Zhu N Zhang J Wang W Li X Yang B Song J. A novel coronavirus from patients with pneumonia in China, 2019. N Engl J Med. (2020) 382:727–33. doi: 10.1056/NEJMoa2001017, PMID: 31978945 PMC7092803

[2] Hassan SA Sheikh FN Jamal S Ezeh JK Akhtar A. A Review of Clinical Features,Diagnosis, and Treatment. Cureus. (2020) 12:e7355. doi: 10.7759/cureus.7355 32328367 PMC7170025

[3] WHO. Coronavirus disease (COVID-19) situation report −132. (2020).

[4] CDC. COVID-19: What you need to know. (2020).

[5] NIH. COVID-19 Research. (2020).

[6] Bao C Liu X Zhang H Li Y Liu J. Coronavirus disease 2019 (COVID-19) CT findings: a systematic review and meta-analysis. J Am Coll Radiol. (2020) 17:701–9. doi: 10.1016/j.jacr.2020.03.006, PMID: 32283052 PMC7151282

[7] Landini N Orlandi M Fusaro M Ciet P Nardi C Bertolo S. The role of imaging in COVID-19 pneumonia diagnosis and manage-ment: main positions of the experts, key imaging features and open answers. J Cardiovas Echogr. (2020) 30:25. doi: 10.4103/jcecho.jcecho_59_20, PMID: 33489733 PMC7811702

[8] Sarkodie BD Mensah YB. CT scan chest findings in symptomatic COVID-19 patients: a reliable alternative for diagnosis. Ghana Med J. (2020) 54:97–9. doi: 10.4314/gmj.v54i4s.14, PMID: 33976447 PMC8087365

[9] Rubin GD Ryerson CJ Haramati LB Sverzellati N Kanne JP Raoof S. The role of chest imaging in patient management during the COVID-19 pandemic: a multinational consensus statement from the Fleischner society. Radiology. (2020) 296:172–80. doi: 10.1148/radiol.2020201365, PMID: 32255413 PMC7233395

[10] Li L Yang L Gui S Pan F Ye T Liang B. Association of clinical and radiographic findings with the outcomes of 93 patients with COVID-19 in Wuhan. China Theranostics. (2020) 10:6113–21. doi: 10.7150/thno.46569, PMID: 32483442 PMC7255034

[11] Li LQ Huang T Wang YQ Wang ZP Liang Y Huang TB. COVID-19 patients' clinical characteristics, discharge rate, and fatality rate of meta-analysis. J Med Virol. (2020) 92:577–83. doi: 10.1002/jmv.25757, PMID: 32162702 PMC7228329

[12] Fang Y Zhang H Xie J Lin M Ying L Pang P. Sensitivity of chest CT for COVID-19: comparison to RT-PCR. Radiology. (2020) 296:E115–7. doi: 10.1148/radiol.2020200432, PMID: 32073353 PMC7233365

[13] Wu J Wu X Zeng W Guo D Fang Z Chen L. Chest CT findings in patients with coronavirus disease 2019 and its relationship with clinical features. Investig Radiol. (2020) 55:257–61. doi: 10.1097/RLI.0000000000000670, PMID: 32091414 PMC7147284

[14] Yang B Bao W Wang J. Active disease-related compound identification based on capsule network. Brief Bioinform. (2022) 23:bbab462. doi: 10.1093/bib/bbab462, PMID: 35057581 PMC8690041

[15] Conceição CCS Martins CM Medeiros Silva M Neto HCCF Chiumello D Rocco PRM. Predicting clinical outcomes at hospital admission of patients with COVID-19 pneumonia using artificial intelligence: a secondary analysis of a randomized clinical trial. Front Med (Lausanne). (2025) 12:1561980. doi: 10.3389/fmed.2025.1561980, PMID: 40385586 PMC12081340

[16] Yang R Li X Liu H Zhen Y Zhang X Xiong Q. Chest CT severity score: an imaging tool for assessing severe COVID-19. Radiol Cardiothorac Imaging. (2020) 2:e200047. doi: 10.1148/ryct.2020200047, PMID: 33778560 PMC7233443

[17] Pennisi M Kavasidis I Spampinato C Schinina V Palazzo S Salanitri FP. An explainable AI system for automated COVID-19 assessment and lesion categorization from CT-scans. Artif Intell Med. (2021) 118:102114. doi: 10.1016/j.artmed.2021.102114, PMID: 34412837 PMC8139171

[18] Chaganti S Grenier P Balachandran A Chabin G Cohen S Flohr T. Automated quantification of CT patterns associated with COVID-19 from chest CT. Radiology. Artif Intell. (2020) 2:e200048. doi: 10.1148/ryai.2020200048, PMID: 33928255 PMC7392373

[19] Shiri I Salimi Y Pakbin M Hajianfar G Avval AH Sanaat A. COVID-19 prognostic modeling using CT radiomic features and machine learning algorithms: analysis of a multi-institutional dataset of 14, 339 patients. Comput Biol Med. (2022) 145:105467. doi: 10.1016/j.compbiomed.2022.105467, PMID: 35378436 PMC8964015

[20] Sun Z Zhang N Li Y Xu X. A systematic review of chest imaging findings in COVID-19. Quant Imaging Med Surg. (2020) 10:1058–79. doi: 10.21037/qims-20-564, PMID: 32489929 PMC7242306

[21] Guan X Yao L Tan Y Shen Z Zheng H Zhou H. Quantitative and semi-quantitative CT assessments of lung lesion burden in COVID-19 pneumonia. Sci Rep. (2021) 11:5148. doi: 10.1038/s41598-021-84561-7, PMID: 33664342 PMC7933172

[22] Kaya F Konya PŞ Demirel E Demirtürk N Orhan S Ufuk F. Visual and quantitative assessment of COVID-19 pneumonia on chest CT: the relationship with disease severity and clinical findings. Current medical. Imaging. (2021) 17:1142–50. doi: 10.2174/1573405617666210215142528, PMID: 33588737

[23] Brouqui P Amrane S Million M Cortaredona S Parola P Lagier J-C. Asymptomatic hypoxia in COVID-19 is associated with poor outcome. Int J Infect Dis. (2021) 102:233–8. doi: 10.1016/j.ijid.2020.10.067, PMID: 33130200 PMC7604151

[24] Somers VK. Kara T. Xie J. ProgressiveHypoxia: A Pivotal Pathophysiologic Mechanism of COVID-19 Pneumonia. Mayo Clin Proc. (2020) 95:2339–2342. doi: 10.1016/j.mayocp.2020.09.015 33153625 PMC7524673

[25] Li K Fang Y Li W Pan C Qin P Zhong Y. CT image visual quantitative evaluation and clinical classification of coron-avirus disease (COVID-19). Eur Radiol. (2020) 30:4407–16. doi: 10.1007/s00330-020-06817-6, PMID: 32215691 PMC7095246

[26] Ko H Chung H Kang WS Kim KW Shin Y Kang SJ. COVID-19 pneumonia diagnosis using a simple 2D deep learning frame-work with a single chest CT image: model development and validation. J Med Internet Res. (2020) 22:e19569. doi: 10.2196/19569, PMID: 32568730 PMC7332254

[27] Jia L-L Zhao JX Pan NN Shi LY Zhao LP Tian JH. Artificial intelligence model on chest imaging to diagnose COVID-19 and other pneumonias: a systematic review and meta-analysis. Eur J Radiol Open. (2022) 9:100438. doi: 10.1016/j.ejro.2022.100438, PMID: 35996746 PMC9385733

[28] Scapicchio C Chincarini A Ballante E Berta L Bicci E Bortolotto C. A multicenter evaluation of a deep learning software (lung quant) for lung parenchyma characterization in COVID-19 pneumonia. Eur Radiol Exp. (2023) 7:18. doi: 10.1186/s41747-023-00334-z, PMID: 37032383 PMC10083148

[29] Ray PP Majumder P. The potential of ChatGPT to transform healthcare and ad-dress ethical challenges in artificial intelligence-driven medicine. J Clin Neurol (Seoul, Korea). (2023) 19:509–11. doi: 10.3988/jcn.2023.0158, PMID: 37635433 PMC10471548

[30] Nazer LH Zatarah R Waldrip S Ke JXC Moukheiber M Khanna AK. Bias in artificial intelligence algorithms and recommendations for mitigation. PLOS Digit Health. (2023) 2:e0000278. doi: 10.1371/journal.pdig.0000278, PMID: 37347721 PMC10287014

[31] Holzinger A Biemann C Pattichis CS Kell DB. What do we need to build explainable AI systems for the medical domain? arXiv. (2017). doi: 10.48550/arXiv.1712.09923

